# Panton-Valentine Leukocidin Does Play a Role in the Early Stage of *Staphylococcus aureus* Skin Infections: A Rabbit Model

**DOI:** 10.1371/journal.pone.0022864

**Published:** 2011-08-05

**Authors:** Urszula Lipinska, Katleen Hermans, Lieve Meulemans, Oana Dumitrescu, Cedric Badiou, Luc Duchateau, Freddy Haesebrouck, Jerome Etienne, Gerard Lina

**Affiliations:** 1 Department of Pathology, Bacteriology and Avian Diseases, Faculty of Veterinary Medicine, Ghent University, Merelbeke, Belgium; 2 Department of Physiology and Biometry, Faculty of Veterinary Medicine, Ghent University, Merelbeke, Belgium; 3 Centre National de Référence des Staphylocoques, Université Lyon 1, Lyon, France; 4 INSERM U851, IFR128, Lyon, France; 5 Hospices Civils de Lyon, Lyon, France; University of California, San Francisco, United States of America

## Abstract

Despite epidemiological data linking necrotizing skin infections with the production of Panton-Valentine leukocidin (PVL), the contribution of this toxin to the virulence of *S. aureus* has been highly discussed as a result of inconclusive results of in vivo studies. However, the majority of these results originate from experiments using mice, an animal species which neutrophils - the major target cells for PVL - are highly insensitive to the action of this leukocidin. In contrast, the rabbit neutrophils have been shown to be as sensitive to PVL action as human cells, making the rabbit a better experimental animal to explore the PVL role. In this study we examined whether PVL contributes to *S. aureus* pathogenicity by means of a rabbit skin infection model. The rabbits were injected intradermally with 10^8^ cfu of either a PVL positive community-associated methicillin-resistant *S. aureus* isolate, its isogenic PVL knockout or a PVL complemented knockout strain, and the development of skin lesions was observed. While all strains induced skin infection, the wild type strain produced larger lesions and a higher degree of skin necrosis compared to the PVL knockout strain in the first week after the infection. The PVL expression in the rabbits was indirectly confirmed by a raise in the serum titer of anti-LukS-PV antibodies observed only in the rabbits infected with PVL positive strains. These results indicate that the rabbit model is more suitable for studying the role of PVL in staphylococcal diseases than other animal models. Further, they support the epidemiological link between PVL producing *S. aureus* strains and necrotizing skin infections.

## Introduction

*Staphylococcus aureus* is a potent human pathogen causing various medical conditions, from minor skin infections (i.a. pimples, impetigo, abscesses) to life-threatening diseases (i.a. bacteraemia, endocarditis and sepsis) [1]. The capacity of *S. aureus* to evoke such a wide range of clinical manifestations may be attributed to the production of numerous exoproteins. One of them, Panton-Valentine leukocidin (PVL), is a two-component (LukS-PV and LukF-PV), pore-forming toxin with affinity for neutrophils and macrophages - cells responsible for the first line defence against invading bacteria [2]–[5]. Epidemiologically, PVL-producing strains have been linked with necrotizing ‘skin and soft tissues infections’ (SSTI) already in the 1990s [6]–[10]. More recently, the emergence of community-associated methicillin-resistant *S. aureus* (CA-MRSA) strains containing PVL determinants and their association with a rapid growing number of skin infections [11], severe necrotising pneumonia [12] and necrotizing fasciitis [13] raised an interest in the importance of PVL in staphylococcal infections.

One of the first *in vivo* studies evaluating the role of PVL in staphylococcal skin infections showed that intradermal injection of PVL provokes widespread and infiltrated erythema followed by skin necrosis in rabbits [6]. In contrast, subsequent *in vivo* experiments using murine models failed to uniformly prove the PVL contribution to the development of staphylococcal skin infections [14]–[17]. The reason for this inconsistency has been linked to a species-specific susceptibility of the host cells to PVL [18], [19]. Since neutrophils isolated from mice have been reported to be relatively insensitive to the leukotoxic effect of PVL in contrast to human and rabbit cells [2], [18], the latter has been postulated to be a better model for studying the role of PVL [18], [19].

Therefore, we employed a rabbit skin infection model in order to evaluate the contribution of PVL to the pathogenicity of the *S. aureus* USA300 strain, a predominant cause of community-associated staphylococcal infections in Northern America [20], using isogenic PVL positive and negative strains.

## Materials and Methods

### Bacterial strains and culture

To provide optimal conditions for the production of PVL, all strains used in this study were cultivated in CCY medium [3]. LAC is a USA300 pulsotype community-associated methicillin resistant *S. aureus* (CA-MRSA) isolate, LACΔ*pvl* is its isogenic PVL knockout strain constructed as described elsewhere [14]. LUG1515 was obtained by trans-complementation of PVL in the LACΔ*pvl* strain using a pLUG534 plasmid [21]. To support plasmid maintenance, all growth media for the LUG1515 strain were supplemented with chloramphenicol (10 µg/ml).

To prepare the bacterial inoculum, a single colony was propagated in 5 ml of casein hydrolysate and yeast extract medium (CCY) medium with constant shaking at 200 revolutions per minute (rpm) at 37°C. The overnight culture was diluted 1∶200 in CCY, incubated till the mid-exponential growth phase (OD_600_ = 0.8) and harvested by centrifugation. The pelleted bacteria were washed once, resuspended in sterile phosphate buffered saline (PBS) to a concentration of about 2×10^9^ colony-forming units (cfu) per ml and immediately used for inoculation. The exact number of bacteria used was determined by plating out serial dilutions of the inocula.

### Experimental animals

Thirty-three twelve-week-old albino hybrid rabbits of either sex, weighing 2.5–3 kg, were purchased from ILVO (Melle, Belgium). These animals do not carry rabbit-associated high virulence *S. aureus* strains evoking i.a. skin lesions, as was confirmed by the absence of such strains in swab samples taken from nine different body sites from all animals [22], [23]. The rabbits were divided randomly into three groups of eleven animals, which were kept in separate HEPA-filtered rooms with negative pressure and light-dark cycles of 12 h per 12 h. The rabbits were housed individually with water and feed provided *ad libitum*.

### Experimental design

From each animal 4 ml of blood was collected from the marginal ear vein before the onset of the experiment. On the day of the infection, the hair on the right flank of each rabbit was shaved with electric clippers (Wella Contura Hair Clipper, Wella) and the skin was disinfected thoroughly with 70% ethanol (Chem-Lab NV, Zedelgem, Belgium). After evaporation of the ethanol, 50 µl of PBS containing 10^8^ cfu of either the knockout strain LACΔ*pvl*, the PVL producing strain LAC or the complemented strain LUG1515 were injected intradermally in the right flank of the rabbits of the respective groups.

During the following 14 days the behavior of the animals and their rectal temperature were examined daily, and the appearance of the lesions was noted. Width and length of the lesions and the necrotic areas were measured, and their surface was calculated as described earlier [15], using the formula 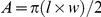, where *A* is a surface, *l* is a length and *w* is a width of the lesion. At day 4 after inoculation (p.i.) three randomly selected animals per group were euthanized. Skin biopsies from the inoculation site were taken aseptically and preserved until further analysis. From the remaining animals, blood samples were collected at day 21 p.i., preceding euthanasia and collection of skin biopsies.

The experimental protocol was approved by the ethical committee of the Faculties of Veterinary Medicine and Bioscience Engineering, Ghent University (permit number 2009_121). The animal studies were performed following the EU legislation being the Council Directive 86/609 EEC covering the protection of animals used for experimental and other scientific purposes. Housing and care of the rabbits was in conformity with the adapted Appendix A of the European Convention ETS 123 of the council of Europe, as approved by the multilateral consultation in 2007.

### Histological analysis

Following fixation in 10% buffered formalin, the skin samples were dehydrated with increasing concentrations of ethanol, cleared with Histosolve (Thermo Shandon, Pittsburgh, PA) and embedded in paraffin. Four microns sections were used to perform hematoxylin-eosin staining. All samples were examined by light microscopy in the absence of any information about their origin.

Hematoxylin-eosin stained sections were evaluated according to the following parameters: degree of dermal necrosis, degree of dermal inflammation and inflammatory cell type, degree of fibrosis and epidermal changes present.

### Detection of anti-PVL antibodies

Blood samples collected during the experiment were incubated for 30 min at room temperature in glass tubes with inert gel barrier and activated disc (Venoject VT109SAS, Terumo). Next, the sera were separated by centrifugation at 1500 rpm for 10 min and stored at −20°C until use.

Antibodies against PVL (before *S. aureus* challenge and at sacrifice) were measured in rabbit sera using an ELISA protocol adapted from Croze et al. [24], with a peroxidase-conjugated swine anti-rabbit polyvalent IgG diluted 1∶10^3^ (DAKO). Serial dilutions of anti-LukS-PV polyclonal rabbit serum (bioMérieux) were used for calibration. The results were expressed in arbitrary units per liter (AU/L), with one arbitrary unit corresponding to the amount of anti-LukS-PV antibodies present in a 1∶10^7^ dilution of the polyclonal rabbit reference serum.

### Statistical analysis

The comparison between strains with respect to diameter of the necrotic area, diameter and surface of the lesion was based on the linear mixed model with strain, time and their interaction as categorical fixed effects and rabbit as random effect. A global significance level of 5% was used. For multiple comparisons (the comparison of the 3 strains at each of the 14 times separately), Bonferroni's adjustment technique was used leading to a comparisonwise significance level of 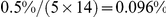.

Antibody levels were compared pairwise between strains at 0 and 21 days after infection using a t-test.

## Results

### Clinical observations

The rectal temperature registered for all rabbits during the experiment was within the normal range [25], with an exception of the first day after the infection. On that day, two animals in group LACΔ*pvl* and one animal in group LUG1515 developed fever (above 40.6°C).

Despite the developing lesions and the raised temperature on day 1 p.i., all animals remained alert, showed normal grooming behavior and appetite throughout the entire experiment.

### Macroscopic description of the lesion development

**Figure 1:**
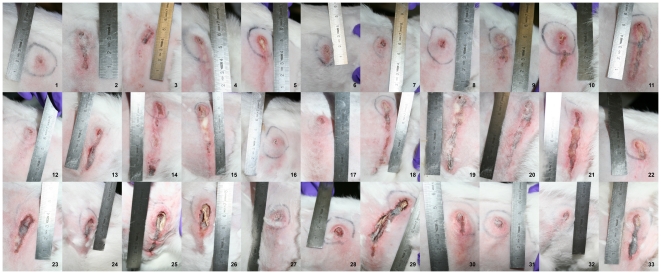
The appearance of lesions on day 4 after infection. The appearance of the lesions on day 4 p.i. in rabbits infected with LACΔ*pvl* (1–11), LAC (12–22) and LUG1515 (23–33). The unit of length of used measuring rod is centimetre.

All 33 rabbits developed an inflammatory reaction at the site of injection of the bacteria within the first 24 h, which within the following days evolved into fully grown lesions. The lesions appeared as a central necrotic area surrounded by a region of inflamed skin. The areas of necrosis became encrusted in all animals by day 5 p.i. (photos in fig. 1 show the appearance of the lesions on day 4). The crusts in the LUG1515 group were situated deeper in the surrounding inflamed skin (fig. 1C), which was not the case in the remaining two groups. Periodic loss of the crusts in all groups revealed purulent material, whereafter a new crust was formed within the following 24 h. Before the end of the observation period (day 14 p.i.) the purulent material within the lesions was replaced by fibrotic scarring tissue.

**Figure 2:**
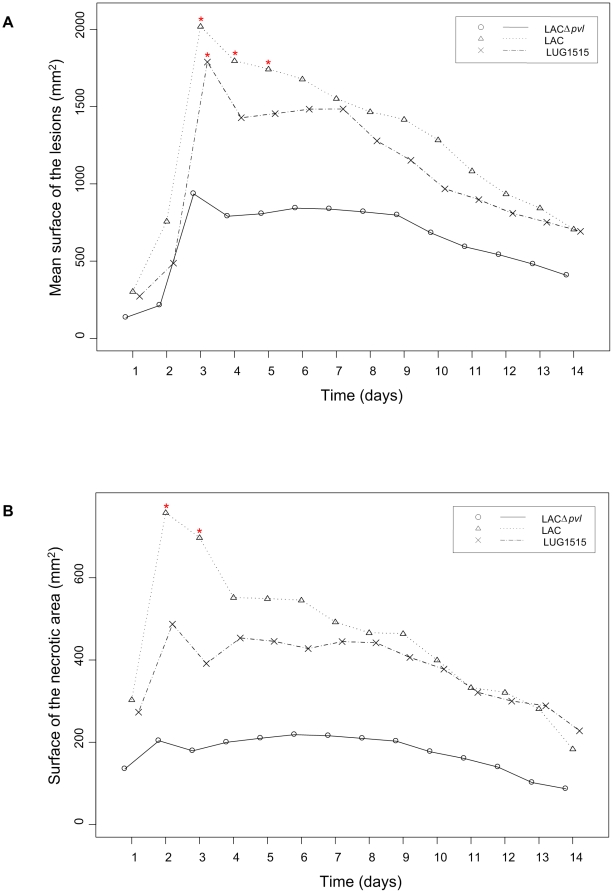
The size of the lesions and skin necrosis over the time. Mean surface of the lesions (a) and necrotic areas (b) evoked by intradermal injection of LAC (Δ), LACΔ*pvl* (o) and LUG1515 (x) in the rabbits. Asterisk (*) represents a statistically significant result in comparison to LACΔ*pvl* group.

A graphic overview of the mean surface of the lesions and necrotic areas in different groups during 14 days after infection is presented in figure 2.

The maximal mean diameter of the lesions was observed for all three infection groups on day 3 p.i. and reached 41 (range 7–75), 80 (range 38–115) and 58 mm (range 25–90) in group LACΔ*pvl*, LAC and LUG1515, respectively. On the last day of the observation the mean diameter of the lesions had decreased to 22 mm (range 10–38) in group LACΔ*pvl*, 35 mm (range 15–55) in group LAC and 30 mm (range 16–63) in group LUG1515. The mean diameter of the lesions in group LACΔ*pvl* was significantly smaller than in group LAC from day 3 till 6 p.i. (P<0.00096). As the surface of the lesions is a function of their diameter, a similar tendency was observed for the mean surface of the lesions, where a significant difference was observed between group LACΔ*pvl* and LAC from day 3 till 5 p.i. The difference between the surface of the lesions of group LACΔ*pvl* and LUG1515 was significant only on day 3 p.i.

The maximal mean diameter of the necrotic area was observed in group LACΔ*pvl* on day 6 p.i., in group LAC on day 2 and in group LUG1515 on day 5, reaching a diameter of 20 (range 7–47), 47 (range 6–92) and 30 mm (range 9–69), respectively. On the last day of the observation the mean diameter of the dermonecrosis had decreased to 12 mm in group LACΔ*pvl*, 23 in group LAC and 17 in group LUG1515. The mean diameter of the necrotic area in group LACΔ*pvl* was significantly smaller than in group LAC on day 2 p.i. (P<0.00096). For days 3–7 p.i. the results were similar, but the difference was not significant. The surface of the dermonecrosis showed a similar tendency with significant difference between LACΔ*pvl* and LAC on day 2 and 3 p.i., and similar but not significant results for days 4–6 p.i.

In the course of the experiment one animal in group LACΔ*pvl*, three in group LAC and two in group LUG1515 developed a secondary lesion on their abdomen. This was possibly a result of gravity-enhanced transport of the bacteria through the lymphatic system. Those lesions were not included in the statistical analysis as there was no physical connection of those lesions with the lesion at the original inoculation site.

### Histological examination

**Figure 3:**
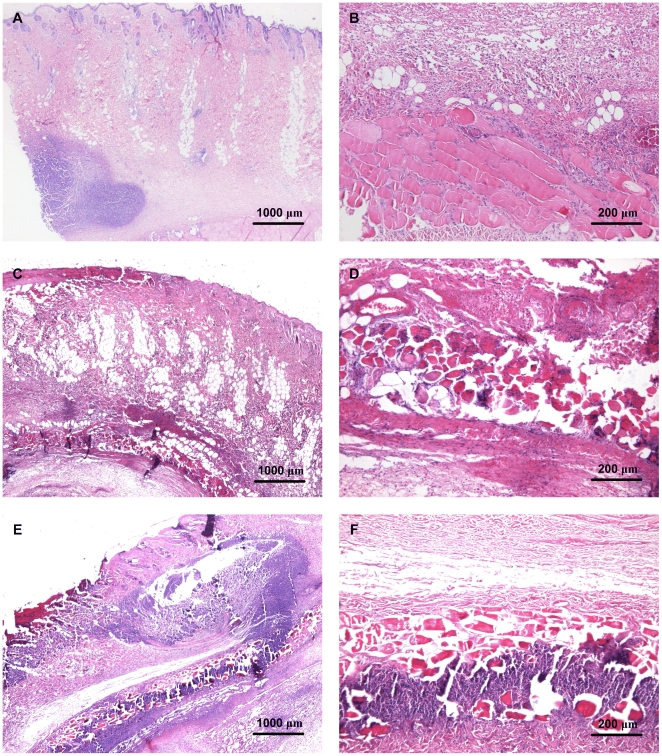
Microscopic appearance of skin lesions on day 4 after infection. The sections of the skin lesions collected from rabbits euthanized at day 4 p.i. were stained with hematoxylin and eosin, and observed under light microscope. A, C and E present the sections from the skin of rabbits infected with LACΔ*pvl*, LAC and LUG1515, respectively. B, D and F show the appearance of the skin muscle layer in animals inoculated with the strains mentioned above.

The histopathological changes observed in skin samples collected from animals euthanized at day 4 p.i. are presented in table 1, and photos of the representative histological sections are shown in figure 3.

**Table 1:** Histopathological changes in rabbit skin produced by PVL-positive or -negative *S. aureus* strains.

Rabbit belonging to group	Necrosis		Inflammation			Remarks
	location	level	location	level	cell type	
LACΔ*pvl*	dermis	++	dermis	++	PMN ++, MF +/−, LF +/−	
	muscle	−	muscle	+	PMN +/−, MF +	
LACΔ*pvl*	dermis	+++	dermis	+++	PMN +++, MF +/−, LF +/−	
	muscle	−	muscle	+	PMN +/−, MF +	
LACΔ*pvl*	dermis	+++	dermis	+++	PMN +++, MF +, LF +	
	muscle	−	muscle	+/−	PMN +/−, MF +	
LAC	dermis	++++	dermis	++++	PMN ++++, MF +, LF +/−	bacteria observed
	muscle	+++	muscle	+++	PMN ++, MF +	
LAC	dermis	+++++	dermis	++	PMN ++, MF +	bacteria observed
	muscle	+++	muscle	+++	PMN +++	
LAC	dermis	+++++	dermis	++++	PMN +++, MF +/−, LF +/−	
	muscle	+	muscle	++	PMN +/−, MF +	
LUG1515	dermis	++++	dermis	++++	PMN +++, MF +, LF +	bacteria observed
	muscle	+++	muscle	++	PMN ++	
LUG1515	dermis	++	dermis	+++	PMN +++, MF +/−, LF +	bacteria observed
	muscle	+	muscle	+	PMN +/−, MF +	
LUG1515	dermis	+++	dermis	++++	PMN +++, MF +, LF +	
	muscle	++	muscle	+	PMN +	

From animals euthanized at day 4 p.i. lesions samples were used to prepare histological slides and the histopathological changes observed are summarized in the table. The grading system used was as follows: for necrosis (−) absent, (+) mild, (++) mild to moderate, (+++) moderate, (++++) pronounced, (+++++) severe; for inflammation (+/−) several inflammatory cells present, (+) mild, (++) moderate, (+++) pronounced and (++++) severe inflammation; presence of specific cell populations (+/−) few cells present, (+) several, (++) some, (+++) many cells present, (++++) big population of cells observed. Abbreviations: PMN = Polymorphonuclear cells; MF = macrophages; LF = lymphocytes.

For rabbits euthanized at the end of the experiment (day 21 p.i.), the following histological findings were noted in the skin. In all samples from the LACΔ*pvl* and LAC inoculated groups there was a marked epidermal hyperplasia and the dermis showed moderate to marked fibrosis with mild perivascular infiltrations of mainly monocytes and lymphocytes. Similar findings were noted in the LUG1515 group, except for one biopsy where there was only a mild dermal fibrosis. In three biopsies from each group some PMNs were present as well. In four LAC, two LUG1515 and one LACΔ*pvl* inoculated skin samples, macrophages were noted. In one biopsy from the LUG1515 group, an old organised intravascular thrombus was present.

### Detection of anti-PVL antibodies in the sera

**Figure 4:**
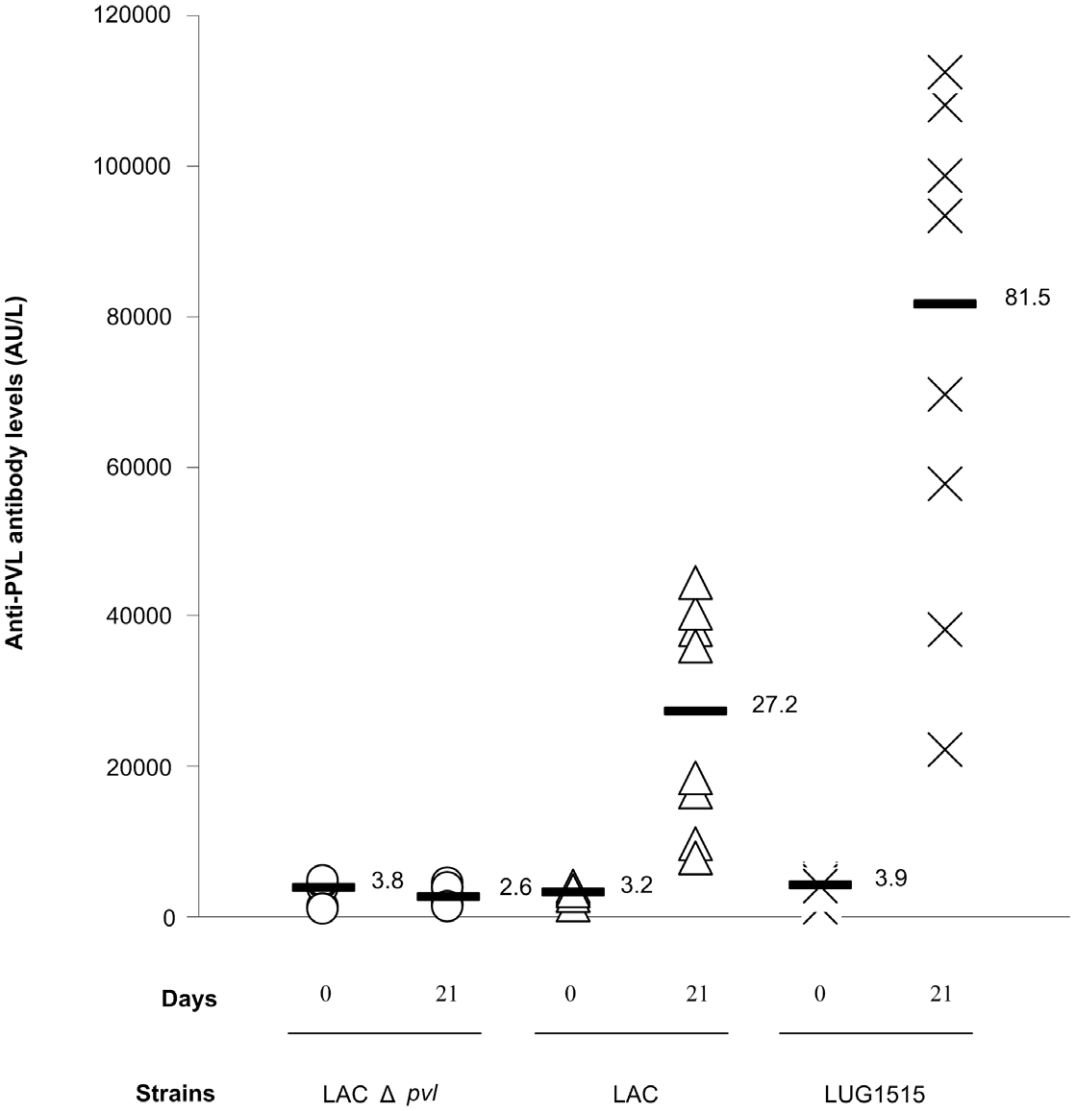
Production of anti-PVL antibodies. Distribution of anti-PVL antibody levels in the sera collected before challenge and at day 21 p.i. from rabbits infected with LACΔ*pvl*, LAC and LUG1515. Bars show the mean value.

The distribution of the titers of the anti-PVL antibodies detected in the sera collected from the rabbits from LACΔ*pvl*, LAC and LUG1515 group before challenge and at euthanasia are presented in figure 4.

There was no statistical difference between the groups in the titer of anti-PVL antibodies in the sera collected before challenge. While in group LACΔ*pvl* there was no statistical difference in the level of antibody before infection and at day 21 p.i., in group LAC and LUG1515 the mean antibody titers were significantly higher in the samples collected at the end of the experiment (8.4 and 19.6 times, respectively).

## Discussion

Involvement of PVL in the virulence of *S. aureus* has been a controversial topic for many scientists searching for factors responsible for the high pathogenicity of community associated *S. aureus* strains. Inasmuch as murine models have been delivering inconclusive results on the PVL contribution to the staphylococcal skin infections, we examined the role of this toxin using a rabbit - an animal species whose neutrophils are as sensitive to the action of PVL as those of humans [18]. We found that the CA-MRSA USA300 isolate expressing PVL produced lesions with significantly bigger surface than the isogenic PVL knockout strain in the first week after inoculation. Those data suggest that PVL has an impact on the early development of *S. aureus* skin infections in rabbits. In line with our results are the conclusions drawn from other studies investigating the role of PVL in rabbit models. Diep et al. depicted a modest, but measurable role of PVL in the pathogenesis during the early stages of bacteremic seeding in the kidney [26], and Cremieux et al. demonstrated PVL contribution to the local extension of osteomyelitis during the early phase of bone infection [27]. With regard to skin infections, our results are the first showing the capability of PVL producing strain to produce bigger lesions in rabbits, in comparison to their isogenic PVL knockout.

Recently, Li et al. published a study showing that rabbits infected with another USA300-type isolate produced lesions with a surface corresponding to that observed in our study in animals infected with LAC [28]. However, in the same study a PVL negative USA500 isolate evoked comparable lesions, which prompted the authors to the conclusion that PVL does not contribute to the development of skin lesions. The variation between the conclusions of the above mentioned and our study could be attributed to the study set up. While strains used in our study varied only upon PVL expression, those used by Li et al. may differ in respect to other determinants as well. Moreover, the latter failed to detect any PVL mRNA by qRT-PCR in the abscesses of PVL positive strains in their experimental model.

As the PVL determinants have been linked with necrotizing infections, the development of skin necrosis was evaluated during our study. The animals in all experimental groups initially developed necrotic lesions in the place of injection of bacteria. Interestingly, the majority of animals inoculated with the PVL knockout strain developed skin necrosis restricted to the surrounding of the inoculation spot. In contrast, the animals injected with PVL producing strains (LAC and LUG1515) developed necrotic lesions largely exceeding the inoculation area, suggesting that PVL activity accounts for development of severe necrotizing lesions. Obtained results are consistent with those of Cribier et al. [6] who observed development of skin necrosis in rabbits injected with purified PVL and observations from a rabbit model of necrotizing pneumonia where a PVL-producing strain caused more lung necrosis than its PVL knockout [19]. Moreover, the histological analysis of skin biopsies collected at day 4 p.i. from the inoculation spot revealed slightly more pronounced damage and inflammatory response in animals infected with PVL positive *S. aureus* strains. The fact that some necrosis and inflammation was observed in the group infected with the PVL knockout strain indicates that other staphylococcal factors (i.e. phenol-soluble modulin, α-toxin) may contribute to the lesion development [28], [29]. In conclusion, the presented results endorse the epidemiological link between PVL-producing strains and necrotizing skin infections [6]–[8], [10].

Interestingly, while only minor histopathological differences have been noted for the dermis, necrosis of the skin muscle layer was observed for animals infected with PVL positive strains, in contrast to the PVL knockout where normal muscle was observed. Similarly, Tseng at al. observed damage of the muscle underlying inoculation spot in mice infected with high doses of PVL producing *S. aureus* [16]. Those data indicate that PVL may contribute to the damage of both the dermis and the skin muscle layer during skin infection.

To confirm the fact that differences between the wild type CA-MRSA strain and its PVL knockout could be accounted to PVL action, our study included a PVL knockout strain expressing PVL from a plasmid (LUG1515). Although the lesions produced by that strain differed macroscopically (smaller, but deeper - see fig. 1 and fig. 2a) from those evoked by the wild type strain, both strains produced bigger lesions in the early phase of infection than the PVL negative strain. Despite the fact that the PVL genes were accompanied by the original promoter and translator, the difference in the structure of the lesions produced by the wild type and the complemented strain could be result of a difference in the amount of PVL produced.

In order to confirm the *in vivo* expression of PVL, blood samples collected from the rabbits were analysed for the presence of antibodies directed against PVL. The anti-PVL antibody level in the rabbits infected with the PVL positive strains increased significantly in the course of the infection, while no shift in the titer of the antibody was observed for the rabbits infected with the PVL knockout strain. This raise in the titer of anti-PVL antibody is an indirect proof for production of this toxin during the infection. In line with our results are the observations from human patients where the anti-PVL antibody titers were demonstrated to be higher in patients with PVL positive *S. aureus* infection and to rise 4.6 times in the course of a skin infection [24].

Our results indicate that the rabbit is a better model than the mouse to study the contribution of PVL in the pathogenesis of skin infections by *S. aureus*. It is important that attention is paid to use animals free of high virulence rabbit *S. aureus* strains [30]. Those can be isolated from different body sites in carrier rabbits and can lead to the development of massive skin infection when introduced into the skin lesion. Through the collection of skin swabs from rabbits prior to the experiment, followed by bacteriological examination and molecular analysis of detected staphylococci, high virulence rabbit *S. aureus* strains can be identified [23] and their interference in the studies of human isolates can be avoided.

Although it could be remarked that the evaluation of the lesions was not performed blinded during this experiment, the significant difference in the size of the lesions evoked by wild type strain and its PVL knockout, in combination with the histological observations, indicate that PVL contributes to the severity of skin infections evoked by the CA-MRSA USA300 strain. As USA300 type isolates are epidemic in the USA and are responsible for a growing number of infections worldwide, further studies concerning community-associated staphylococcal infections should not neglect the impact of PVL.
